# Alcohol and Drug Use Among Injured Drivers: Insights From an Emergency Room Study at an Institute of National Importance in India

**DOI:** 10.7759/cureus.89029

**Published:** 2025-07-30

**Authors:** Kamal Kant Sahu, Swapnil P Akhade, Krishnadutt Chavali, Pankaj S Ghormade

**Affiliations:** 1 Forensic Medicine and Toxicology, All India Institute of Medical Sciences, Raipur, Raipur, IND

**Keywords:** drunk driving, emergency medicine, india, road traffic injuries, substance abuse

## Abstract

Background

Motor vehicle crashes (MVCs) remain a leading global public health challenge, with driving under the influence of psychoactive substances significantly elevating crash risk. Despite India’s high burden of road traffic injuries, data on substance use among injured drivers - particularly in central India - are limited.

Objective

This study aimed to determine the prevalence and patterns of alcohol and drug use among drivers involved in MVCs, presenting to the Emergency Department of a tertiary care hospital in Raipur, India.

Methods

This cross-sectional study was conducted with 170 drivers who were admitted to the Trauma and Emergency Department of a tertiary care centre. The drivers were tested for the presence of various substances, including alcohol, cannabis, benzodiazepines, opioids, amphetamines, cocaine, and barbiturates, using rapid detection kits. Data on demographics, crash characteristics, and substance use were analysed using descriptive statistics and chi-square tests.

Results

Alcohol (45.9%), cannabis (21.9%), and benzodiazepines (9.4%) were the most detected substances, exceeding national averages. Polydrug use was common (14.1%), though benzodiazepines were never detected alone. Urban drivers showed higher substance use rates (cannabis: 59.1%; alcohol: 42.3%; opioids: 16.5%). No female drivers tested positive. Substance use was associated with life-threatening injuries in a significant population (79.2%).

Conclusion

The high prevalence of substance use among injured drivers underscores the need for stricter enforcement of impaired driving laws (e.g., roadside testing) and public health interventions targeting at-risk populations. Multicentric studies are warranted to validate these findings.

## Introduction

Globally, drug-induced impairment in driving is rising. While the dangers of drinking and driving are well-established, there's a need to address other drug-induced impairments. The world faces a longstanding drug issue, and there are knowledge gaps, particularly in connection with effective addiction prevention measures [1]. Different drugs affect driving skills in different ways, depending on how they interact with the brain. The drugged driver puts the driver, passenger, and others who share the road at serious risk [2]. Road traffic deaths are the eighth leading cause of death for those aged 5-29, with 1.35 million deaths in 2016. The African region has the highest mortality rate, followed by South-East Asia [3]. Global drug abuse is a major issue highlighted by the World Drug Report (2021), published by the UN Office on Drugs and Crime. According to the report, approximately 200 million people use cannabis, 20 million use cocaine, about 62 million use opioids for non-medical reasons, and approximately 27 million use amphetamine stimulants [4]. According to the National Crime Records Bureau, 1,73,860 road traffic deaths occurred in India in the year 2021. Drug- or alcohol-related driving accounted for 1.9% of these total accidents and resulted in 2,935 deaths [5]. In the Indian context, according to the National Crime Records Bureau, 1,73,860 people died in road traffic crashes (RTCs) in 2021. In total, 2,935 deaths were caused by drug- or alcohol-related driving [5].

The "Magnitude of Substance Use in India 2019" survey by the National Drug Dependency and Treatment Centre at AIIMS Delhi revealed alcohol (14.6%) as the most commonly abused drug, followed by cannabis (2.8%), opioids (2.62%), sedatives (1.08%), amphetamines (0.18%), and cocaine (0.10%). India’s highest alcohol consumption was found in Chhattisgarh state, followed by Tripura, Punjab, Arunachal Pradesh, and Goa [6].

A review of studies conducted by the Indian researchers from 1980 to 2011 found a correlation between substance abuse and traffic injuries. Psychotropic substances were found in 2%-33% of injured victims and 6%-48% of fatally injured victims. The use of these substances impairs judgment, increasing the likelihood of speeding, risk-taking, and traffic rule violations, thus contributing to RTC involvement [7]. Henceforth, adequate measures against drugged and drunken driving can curtail most RTCs. The persistence of drugged and drunken driving is partly due to inadequate testing facilities, which hinder the identification of drug abusers involved in road accidents. This deficiency in testing also hides the true prevalence and scope of drug abuse among drivers. However, recent advancements in affordable and dependable drug detection kits allow for quick testing of suspected cases, providing results within minutes. It's widely known that accident victims often use drugs, making rapid point-of-collection (POC) devices crucial for gathering epidemiological data on drugged driving prevalence and promptly identifying substance abusers for treatment. Moreover, advanced POC technology has the potential to reduce future drug-related motor vehicle crashes (MVCs) while enhancing overall traffic safety [8].

MVCs account for a substantial portion of the primary reasons for referrals to Trauma and Emergency services. However, there is limited data available in Chhattisgarh state that correlates substance abuse among drivers with these accidents. Therefore, this study was conducted to assess the prevalence and characteristics of drivers involved in MVCs that were reported to the Emergency Department at an Institute of National Importance located in Chhattisgarh, central India.

## Materials and methods

Study population

The study was approved by the Institutional Ethical Committee at AIIMS Raipur under proposal number AIIMSRPR/IEC/2021/785. It included all consenting injured drivers who presented to the trauma centre following road accidents from September 2020 to January 2021, while 170 subjects were selected after careful exclusion of cases according to the study protocol.

Inclusion criteria

The study included drivers older than 18 years who arrived at the Emergency Department within six hours of the accident and were willing to provide written informed consent (consent was obtained from legal guardians for unconscious or unstable patients).

Exclusion criteria

Consecutive sampling was used to select the participants, and the study excluded patients on treatment with opiates or benzodiazepines; those who were unstable or unable to provide a sample, such as patients with hepato-renal impairment; cases lacking documentation of the accident’s time and date; unconscious or intoxicated patients without a legal representative; and those unable to provide a urine sample.

Sample collection and screening

Researchers collected urine samples after obtaining informed consent, testing them for alcohol, benzodiazepines, barbiturates, cannabis, amphetamines, cocaine, and opiates using two rapid drug detection kits: SURESTEP™ Drug Screen Cassette ©2021 (Abbott Laboratories, Abbott Park, IL, USA) for alcohol, and Wondfo® One Step Multi-Drug Urine Test Panel (Guangzhou Wondfo Biotech Co., Ltd., Guangzhou, China) for other substances. The testing procedure involved opening the kit, dipping the strip in urine for 20-30 seconds for the multi-panel test, and adding three drops of urine to the specimen well for the analysis. Results were read after five minutes, and all waste was disposed of according to biomedical waste management guidelines. The test device is an in vitro immunoassay that analyses drugs in urine, claiming over 99% accuracy compared to standard methods. 

Data collection and statistical analysis

Data collected from the respondent and psychotropic substance testing results have been entered into the case record form. The data from the case record form were entered into a Microsoft Excel spreadsheet (Microsoft® Corp., Redmond, WA, USA). Demographic profiles and crash characteristics, as well as positive results for various substances abused, were analysed using descriptive statistics. The Chi-square test was performed using IBM SPSS Statistics for Windows, Version 26 (Released 2019; IBM Corp., Armonk, NY, USA). The significance level was set at p < 0.05, and the confidence interval at 95%. The relationship between substance use and other variables was analysed to determine whether statistically significant differences existed.

## Results

The study analysed 170 injured drivers, comprising 153 males (90%) and 17 females (10%). As described in Table 1, most participants were aged 18 to 31 (55.9%), with only 3.53% aged 60 or above. About 54% of men and 94% of women lived in urban areas. Educationally, 65.5% had only completed school, and 26.5% were college graduates, predominantly female. Employment-wise, 28.8% were unemployed (including housewives and students), 26.5% were self-employed, and only 20.6% worked in public or private sectors, with 59% of women unemployed. Commercial drivers (78.6%), self-employed individuals, including farmers (68.9%), and labourers (66.7%) had higher positivity rates for substances compared to unemployed individuals (36.7%) and employees (37.1%). About 55% of unemployed and commercial drivers tested positive for other psychotropic substances, while most self-employed, labourers, and employees showed positive results for alcohol alone. Approximately 53.5% were married. During the accidents, 60% were alone, and only 22.4% wore seat belts or helmets. Additionally, 31.8% reported consuming substances prior to the crash, while 68.2% denied it.

**Table 1:** Frequency distribution of demographic profile of injured driver (N = 170) As depicted, the study included 170 injured drivers: 153 males (90%) and 17 females (10%). Injuries occurred most frequently in individuals aged 18 to 32 years (55.9%), with both males and females predominantly in this age group. Urban areas accounted for 54% of men and 94% of women. High school education was the most common educational level (65.5%), followed by college graduation (26.5%). There were 28.8% unemployed participants (including students and housewives), 26.5% who were self-employed, and only 20.6% who were employed. Among the female participants, 59% were unemployed. The majority of injured drivers in car accidents (60%) were alone, and only 38 of them (22.4%) wore a seat belt.

Sociodemographic profile		Frequency	Percent
Age group (years)	< 18	12	7.1
	18 - <32	95	55.9
	32 - <46	45	26.5
	46 - <60	12	7.1
	60 and >60	6	3.5
Sex	Female	17	10.0
	Male	153	90.0
Address	Rural	72	42.4
	Urban	98	57.6
Education	Illiterate	1	0.6
	School	113	66.5
	Graduates	45	26.5
	Postgraduates	11	6.4
Occupation	Unemployed	49	28.8
	Labourer	27	15.9
	Commercial driver	14	8.2
	Self employed	45	26.5
	Employed	35	20.6
Marital status	Married	91	53.5
	Unmarried	79	46.5
Total		170	100.0

As shown in Tables 2-3, out of 170 participants, 91 individuals (53.5%) tested positive for drug abuse. The most commonly abused substance was alcohol, accounting for 45.9% of cases, followed by cannabis at 12.9% and benzodiazepines at 9.4%. Morphine was detected in 3.5% of the cases, while barbiturates were identified in just one instance. Notably, 14.1% of participants tested positive for two or more substances; among these, 20 out of 24 multi-substance users had also tested positive for cannabis. Within the alcohol-positive group, there were 62 individuals who tested positive solely for alcohol and 16 who tested positive for alcohol along with other substances. The study uncovered a significant gender disparity: over 59.5% of male participants tested positive for substance abuse, whereas no female participants tested positive, indicating that substance abuse was limited to males in this cohort.

**Table 2:** Test results for substance use in urine samples (N = 170) A total of 54 drivers (31.8%) admitted to consuming substances before the incident, while 116 (68.2%) denied it. Of the 170 study participants, 91 (53.5%) showed evidence of drug abuse in their urine.

Test result	Frequency	Percent
Negative	79	46.5
Positive	91	53.5
Total	170	100

**Table 3:** Distribution of various substance and their combinations The table shows that, out of 91 positive results (n = 91), 67 (73.6%) were positive for only one substance, while 24 (26.4%) were positive for two or more substances. Alcohol was the most commonly abused substance (85.71%), either alone or in combination with other substances. Cannabis was found to be the second most commonly abused drug, followed by benzodiazepines. Morphine was detected in 3.5% of cases, and a barbiturate was detected in only one instance. Among the 22 cannabis-positive cases, 99% also tested positive for other substances. THC, tetrahydrocannabinol

Test results		Frequency	Percent
Positive for one substance (67; 73.6%)	Alcohol	62	68.1
	Morphine	3	3.3
	Cannabis (THC)	2	2.2
Positive for more than one substance (24; 26.4%)	Alcohol + Barbiturates	1	1.1
	Alcohol+ Benzodiazepine	1	1.1
	Alcohol + Cannabis (THC)	7	7.7
	Benzodiazepine + Morphine	2	2.2
	Benzodiazepine + Cannabis (THC)	5	5.5
	Alcohol + Benzodiazepine + Cannabis (THC)	7	7.7
	Benzodiazepine + Cannabis (THC) + Morphine	1	1.1
Total		91	100.0

Table 4 shows that most individuals who tested positive for substance abuse were under 30, while those aged 30 to 40 had the highest positivity rate, at 60.8%. Alcohol was the most commonly used substance across all age groups. About 69% of those who tested positive for benzodiazepines were under 30, and a similar trend was seen for tetrahydrocannabinol (THC). Notably, 66.7% of participants who tested positive for morphine were aged 30 to 40.

**Table 4:** Distribution of positively screened individual substance with age of the injured drivers When considering individual substances, alcohol was the most common among all age groups. About 69% of those who tested positive for benzodiazepines were under 30 years old, and the majority of those who tested positive for cannabis were also under 30 years. Most of the participants who tested positive for morphine (66.7%) were between 30 and 40 years of age.

Age (years)	Individual substance screened positive				
	Alcohol, n (%)	Cannabis, n (%)	Benzodiazepine, n (%)	Morphine, n (%)	Barbiturates, n (%)
<30	40 (51.3)	18 (81.9)	11 (68.7)	1 (16.7)	1 (100)
30 - 40	26 (33.3)	3 (13.6)	4 (25)	4 (66.6)	0 (0)
>40	12 (15.4)	1 (4.5)	1 (6.3)	1 (16.7)	0 (0)
Total	78 (100)	22 (100)	16 (100)	6 (100)	1 (100)

As shown in Table 5, 24 victims arrived at the hospital in life-threatening condition, with 19 of them (79.2%) testing positive for substances. In contrast, among the 146 victims in non-life-threatening condition, 72 (49.3%) tested positive - a significant difference (p < 0.01). Light vehicle drivers (bicycles, motorcycles, and cars) had a 53% positive rate for substance abuse, while heavy vehicle drivers (cars and commercial trucks) had a rate of 66.7%. Only 35 participants (20.6%) accepted blame for the accident, with 33 of them testing positive for substances. In comparison, 61 participants blamed factors like adverse weather or traffic for their crashes, and 26 (42.6%) tested positive for drugs. Additionally, 74 participants blamed the other vehicle, with 32 (43.2%) also testing positive for substances. Table 6 illustrates that alcohol was the most common substance of abuse among both light and heavy vehicle drivers. The study findings, mentioned in Table 7, indicate that substance abuse prevalence among vehicle drivers is lowest in Norway and highest in the United States. Research conducted in India revealed that approximately 50% of vehicle drivers are affected by substance use. Additionally, Table 8 shows that the prevalence of alcohol abuse among drivers aligns with the national data on the general population of India.

**Table 5:** Distribution of cases by substance use and health condition at the time of admission The table presents the health status of patients upon admission. It was found that 79% of drivers with life-threatening conditions reported substance use. The association between substance use and life-threatening health conditions is found to be significant.

Participants condition at the time of admission	Test result			p-value (chi-square value)
	Negative	Positive	Total	
Non-life threatening	74	72	146	<0.01 (X^2 ^= 17.617)
	50.7%	49.3%	100.0%	
Life threatening	5	19	24	
	20.8%	79.2%	100.0%	
Total	79	91	170	
	46.5%	53.5%	100.0%	

**Table 6:** Distribution of cases by substance use and type of vehicle used by the participant The data presented indicates that the prevalence of substance abuse among drivers varies between light vehicle drivers (including motorcycles and automobiles) and heavy vehicle drivers (such as trucks). Specifically, 53% of light vehicle drivers tested positive for substance abuse, while the percentage for heavy vehicle drivers was 66.7%. In both categories of drivers, alcohol emerged as the most commonly abused substance, followed by cannabis.

Drivers occupancy	Test result					p-value
	Negative		Positive	Total		
Light vehicle (two-wheelers and three-wheelers)	77	87			164	0.511 (X^2 ^= 3.14)
	47%	53%			100.0%	
Heavy vehicle (four-wheelers and above)	2	4			6	
	33.3%	66.7%			100.0%	
Total	79	91			170	
	46.5%	53.5%			100.0%	

**Table 7:** Comparison of prevalence of substance use among various studies conducted throughout the world

Studies	Country	Year	Prevalence (%)
Walsh et al. [8]	Australia	2004	59.3
Drummer et al. [9]	Australia	2012	52
Derakhshanfar et al. [10]	Iran	2012	39
Kumar et al. [11]	India	2015	54.5
Jørgenrud et al. [12]	Norway	2019	4.9
Mundenga et al. [13]	Tanzania	2019	46.9 (alcohol) and 36.1 (drugs)
Tonellato et al. [14]	USA	2021	73.4
Wong et al. [15]	Hong Kong	2010	10
Present study	India	2023	53.5

**Table 8:** Comparison of prevalence of individual substance between present study and national data The table above presents a comparative analysis of the prevalence of individual substance use among injured drivers and among the general population as per national data available on the Ministry of Social Justice and Empowerment's website (Government of India) [6].

Substance use	Prevalence among the general population as per national data (%)	Prevalence among injured drivers in the present study (%)
Alcohol	14.6	45.9
Cannabis	2.8	12.9
Morphine	2.1	3.5
Sedative	1.08	1.0
Amphetamine	0.18	Nil
Cocaine	0.10	Nil

## Discussion

In India, it is illegal to drive with a blood alcohol concentration (BAC) exceeding 30 mg per 100 mL, reflecting a broader recognition of the dangers posed by both alcohol and drug use while driving. With BAC legally capped, there is a global understanding that impairment due to drugs also warrants strict regulation [16]. Indian demographic patterns of RTCs highlight a concerning trend: young male drivers, who comprise the majority of both registered drivers and those involved in RTCs, often exhibit higher rates of substance use prior to driving [17]. Data indicate that young male drivers are disproportionately involved in RTCs, accounting for the majority of fatalities; males represent about 87.3% of all road accident deaths, compared to 12.7% for females [18]. WHO data show that road traffic deaths are higher among men due to greater risk exposure and risk-taking behaviour [17,18]. An extensive review of 46 studies in low- and middle-income countries found that males are involved in 80% of crashes and represent 87% to 100% of drivers [19]. Although females represent only 6.8% of registered drivers in India, their involvement in accidents is relatively low [20].

The findings of our study substantiate this observation. As per the Ministry of Road Transport & Highways report on “Road Accidents in India 2020,” around 70% of fatal road accident victims were between the ages of 18 and 45 [17]. Our study corroborated this national data, with 82% of the injured drivers being between the ages of 18 and 46 years. A total of 64% of female participants were between the ages of 18 and 32 years. There were no female participants under 18 or over 60 in our study. It can be concluded from the above data that RTCs primarily involve young adults. For a variety of reasons, people in this age group are more vulnerable to RTCs.

According to the NCRB 2021 report, 62% of road accidents occur in rural areas, while 40% happen in urban areas [5]. In contrast, our study found that 42% of participants were from rural areas and 58% from urban areas. This difference is likely because many urban residents seek treatment at our national institute, while we mainly receive referrals from remote rural areas. Notably, 94% of the female participants were from urban backgrounds, reflecting greater empowerment and independence among urban women, compared to those in rural settings.

In a study by Kumar et al. in northwestern India, 60% of injured drivers who tested positive for psychoactive substances were under 34 years old, aligning with our findings in Central India. Kumar et al. reported a high percentage of participants aged 18-34 who tested positive for alcohol and opiates, while most cases positive for cannabis and benzodiazepines were in the 55-plus age group, which differs from our study results [11]. Similarly, a study in Australia by Ch’ng et al. found that cannabis use was most common among those aged 15 to 44, with a significant percentage of participants across all age groups testing positive for benzodiazepines and opiates. The findings of our study are more in agreement with those of the Australian study [21]. In terms of alcohol and cannabis use, a recent study from the USA by Derefinko et al. showed that rural students were less likely to use them as beginners than their urban counterparts [22]. According to an article published in the USA by Bunn et al., most fatal crashes were reported from rural areas, but there was no significant variation in alcohol or drug testing of fatally injured drivers between rural and urban areas [23]. According to our study, about two-thirds of participants from rural areas tested positive for substance abuse, compared to 45% of those from urban areas. In terms of individual substances, rural areas accounted for about 58% of alcohol-positive cases. We also found that cannabis-positive cases accounted for 59.1%, benzodiazepine-positive cases for 75%, and opium-positive cases constituted almost 83% of injured drivers. This could probably be due to the greater availability of psychotropic substances in the urban setting. Considering the above findings, alcohol is the main substance abused in rural areas, while in urban areas, it is often used in combination with other drugs. Most participants in this study had formal education (66.5%), with 26.5% being graduates and 6.4% postgraduates. Only one participant was illiterate. In a similar study conducted in India, Farooqui et al. found that 56% of cases were at the junior college level, and 16% were graduate and postgraduate students [24]. Our study found that 8% of participants were unemployed, 26.5% were self-employed, 20.6% worked in service, 16% worked as labourers, and 8.2% worked as commercial drivers. The unemployed category also included students and housewives. Self-employed individuals included those who ran their own businesses. The study by Farooqui et al. reported findings that were consistent with our study [24].

Iranian researchers Divsalar et al. [25] found a significant correlation between substance use and low education levels in a study conducted in a similar setting. We find this study to be in accordance with our findings. According to the epidemiological study by Ghulam et al. [26], with literacy rates rising, abuse rates increase as well. The authors explained that illiterate and low-income groups have less purchasing power compared to literate and high-income groups. In contrast, the lower incidence of substance use among highly educated individuals may be due to increased awareness about the harmful effects of substance use [26]. In our study, 14% of participants were in a life-threatening condition. Among those with non-life-threatening injuries, 49.3% tested positive for substance abuse, while 79.2% of participants with life-threatening conditions tested positive for psychotropic substances, showing a statistically significant difference between the groups. According to Odoardi et al., drug abuse was more prevalent among fatally injured drivers than non-fatally injured drivers [27]. This is consistent with our study findings. The use of psychotropic substances is harmful to an individual's health and destroys the harmony of the family. In addition, it damages the social and economic status of both the family and society. Driving under the influence of these substances can lead to injury and death for the driver, as well as for other road users, and this, in turn, can cause an economic burden on society.

Recommendations

Although Indian law prohibits the use of alcohol and drugs while driving, drug testing is rarely conducted. India penalises drunk drivers through roadside breath alcohol testing; however, no protocols are in place to screen for other psychotropic drugs. Further research involving quantitative analyses of substances abused by drivers involved in vehicle crashes is needed.

Limitations of the study

Psychotropic substances were only screened for in injured drivers, without a control group. Therefore, it is difficult to determine whether injured drivers use psychotropic substances more frequently than the general population. Testing positive for a drug does not necessarily indicate driving impairment, as the time and dose of drug intake may have occurred many hours before urine sample collection. Some drug screening tests detect inactive metabolites of abused drugs, and cross-reactivity can produce false positives in immunoassays.

## Conclusions

The overall prevalence of psychotropic substances remains high in the present study. Alcohol was the most commonly abused substance (85.71%), either alone or in combination with other substances. Cannabis was found to be the second most commonly abused drug, followed by benzodiazepines. Compared to the national average, alcohol, cannabis, and benzodiazepines were more prevalent in our study. The number of cases in which more than one substance was detected was significant. None of the female participants tested positive for psychotropic substances. Cannabis, benzodiazepines, and opiates were more prevalent in urban areas. In most participants with life-threatening injuries reported to trauma centres, psychotropic substances were detected. Considering these findings, objective methods of identifying psychotropic substance abuse in drivers are urgently needed. The existing laws for the prosecution of this offence need to be updated as well.
